# Mitochondria Related Cell Death Modalities and Disease

**DOI:** 10.3389/fcell.2022.832356

**Published:** 2022-03-07

**Authors:** Chuwen Tian, Yifan Liu, Zhuoshu Li, Ping Zhu, Mingyi Zhao

**Affiliations:** ^1^ Department of Pediatrics, The Third Xiangya Hospital, Central South University, Changsha, China; ^2^ Xiangya School of Medicine, Central South University, Changsha, China; ^3^ Guangdong Cardiovascular Institute, Guangdong Provincial People’s Hospital, Guangdong Academy of Medical Sciences, Guangzhou, China

**Keywords:** regulated cell death, mitochondria, mitochondrial diseases, ferroptosis, parthanatos

## Abstract

Mitochondria are well known as the centre of energy metabolism in eukaryotic cells. However, they can not only generate ATP through the tricarboxylic acid cycle and oxidative phosphorylation but also control the mode of cell death through various mechanisms, especially regulated cell death (RCD), such as apoptosis, mitophagy, NETosis, pyroptosis, necroptosis, entosis, parthanatos, ferroptosis, alkaliptosis, autosis, clockophagy and oxeiptosis. These mitochondria-associated modes of cell death can lead to a variety of diseases. During cell growth, these modes of cell death are programmed, meaning that they can be induced or predicted. Mitochondria-based treatments have been shown to be effective in many trials. Therefore, mitochondria have great potential for the treatment of many diseases. In this review, we discuss how mitochondria are involved in modes of cell death, as well as basic research and the latest clinical progress in related fields. We also detail a variety of organ system diseases related to mitochondria, including nervous system diseases, cardiovascular diseases, digestive system diseases, respiratory diseases, endocrine diseases, urinary system diseases and cancer. We highlight the role that mitochondria play in these diseases and suggest possible therapeutic directions as well as pressing issues that need to be addressed today. Because of the key role of mitochondria in cell death, a comprehensive understanding of mitochondria can help provide more effective strategies for clinical treatment.

## Introduction

With advancements in mitochondrial research, multiple functions of mitochondria have received increased attention. Mitochondria are the energy metabolism centre of eukaryotic cells. The common pathway of final oxidation that mitochondria are responsible for is the tricarboxylic acid cycle and electron transport chain, which correspond to the second and third stages of aerobic respiration, respectively (van der Bliek et al., 2017). Mitochondria play an important role in maintaining cell homeostasis. The level of potassium ions in the mitochondria affects the level of oxidative stress. The newly discovered mitoKATP channel has been found to regulate mitochondria in response to cellular stress by regulating the volume of the matrix (Paggio et al., 2019). Moreover, research suggests that different nuclear-mitochondrial combinations affect metabolism in different ways. A recent study found that mitochondrial fission factor is related to voltage-dependent anion channel-1 on the outer mitochondrial membrane *in vivo*. The mitochondrial fission factor and voltage-dependent anion channel-1 complex triggers a variety of cell death mechanisms (Seo et al., 2019). Mitochondrial calcium uniporter (MCU), which is the mitochondrial inner membrane transporter, can also maintain mitochondrial calcium homeostasis by absorbing calcium ions (Oxenoid et al., 2016). Lambert et al. (Lambert et al., 2019) discovered a protein known as MCUB, which is similar to MCU. The MCUB gene regulates the opening of calcium channels on the cell membrane, and its gene expression is increased in mice with heart diseases, while the expression levels of MCU and the channel MICU1 are decreased. In addition, Qian et al. (Qian et al., 2019) link mitochondria to telomere function. The hydrogen peroxide produced by mitochondria enters the nucleus and destroys telomere function but does not cause overall damage to the nuclear DNA.

The Nomenclature Committee on Cell Death states that cell death is divided into two distinct forms, accidental cell death and regulated cell death (RCD). RCD is a genetic coding mechanism in multicellular and unicellular eukaryotes whose function is to maintain tissue and organism homeostasis by removing needless cells and irreversibly damaged, abnormal or potentially harmful cells during development. This kind of regulated cell death includes apoptosis, pyroptosis, autophagy, ferroptosis and alkaliptosis (Galluzzi et al., 2018). Mitochondria are highly plastic organelles that constantly change their shape and size through fusion and division processes (collectively referred to as mitochondrial dynamics) in response to metabolic and signalling cues in the cellular environment (Mishra and Chan, 2014).

**FIGURE 1:**
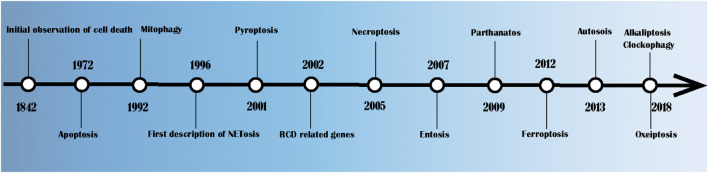
Timeline of cell death related research.

As shown in Figure 1, Karl Vogt first observed the natural death of cells in crickets in 1842 (Kerr et al., 1972). In 1972, John Kerr, Andrew Wyllie and Alastair Currie formally put forward the term “apoptosis” and pointed out that it is a method of cell death whose morphological characteristics are different from those of classical cell necrosis (Kerr et al., 1972). Since then, there has been a great deal of research on RCD. In 1992, Ohsumi clarified the potential mechanism of autophagy in yeast, for which he won the 2016 Nobel Prize in Physiology and Medicine (Okamoto et al., 2009). In 1996, studies found that caspase maintains balance by regulating cell death and inflammation (McIlwain et al., 2013). NETosis was first described in the same year (Takei et al., 1996). The concept of pyroptosis, that is, the procedural necrosis that relies on caspase-1, was formally proposed in 2001 (Shi et al., 2017). Necroptosis was first described in 2005 in studies of cerebral ischaemia reperfusion (Degterev et al., 2005). In 2007, Overholtzer, M. et al. (Overholtzer et al., 2007) discovered a new method of cell death that is known as entosis. In this process, one or more living cells enter the cytoplasm of another cell, forming a cell overlapping structure and leading to cell death. Dawson, V. L. first proposed the concept of parthanatos in 2009 (David et al., 2009). In 2012, Dixon et al. proposed iron-dependent RCD, which is known as ferroptosis (Dixon et al., 2012). In 2013, Liu et al. (Liu et al., 2013) revealed a novel autophagy-dependent cell death, which is known as autosis, which is regulated by Na^+^, K^+^—ATPase. In 2018, DaolinTang et al. demonstrated that JTC801 specifically induced pH-dependent cell death of cancer cells by reducing the expression of CA9. Since then, the concept of alkaliptosis was proposed (Song et al., 2018). In 2018, Holze et al. (Holze et al., 2018) proposed a caspase-independent cell death induced by reactive oxygen species (ROS), which is known as oxeiptosis.

Mitochondrial-regulated cell death plays an important role in human diseases. It not only plays a role in the pathogenesis of many diseases but also provides ideas for their treatment. We have summarised the role of mitochondria in diseases of different systems in Table 1. The positive aspects and negative aspects of various cell death forms in mitochondrial diseases are shown in Table 2. In this review, we discuss how mitochondria participate in various cell death modes, as well as the basic research and the latest clinical progress on mitochondria in various organ system diseases. Based on the key role of mitochondria in cell death, a better understanding of mitochondria can help provide more effective strategies for clinical treatment.

**TABLE 1:** The role of mitochondria in diseases of different systems.

Diseases	Mitochondrial related targets	References
Nervous System	Delays Aβ-induced paralysis ameliorates depletion of the mitochondrial lipid cardiolipin protects complexes IV and I of the ETC.	Ng et al. (2014)
	Inhibit BCAT-1 (RNAi) expression	Mor et al. (2020)
	Increase NAD+ and NADH levels	Schondorf et al. (2018)
	Reduce ROS levels calcium overload and deposition of Aβ1-42	Hu et al. (2018); Wang et al. (2019a)
	Inhibit high expression levels of Bax, Bad, and cleaved caspase-3 and caspase-8	Li et al. (2017); Hu et al. (2018)
	Inhibit Drp1 expression	Manczak et al. (2016)
	Inactivated NLRP3	Wang and Ge (2020)
Respiratory System	TRPA1 and TRPV1 channels	Wang et al. (2019b)
	Improve PRKN level	Araya et al. (2019)
	NRF2	Boutten et al. (2011)
Endocrine Diseases	Activating PDH	Perry et al., 2015
	Mitochondrial uncoupling	Nedergaard et al. (2005)
	DCA combined with DNP preferentially induced glucose oxidation	Erion et al. (2013)
Urinary System	CoQ transmits electrons and antioxidants	Doimo et al. (2014); Opalińska and Meisinger (2015)
	miR-21 significantly up-regulated	McClelland et al. (2015)
	Epigenetic regulation by miR-93	Zhong et al. (2013)

**TABLE 2:** The positive aspects and negative aspects of various cell death forms in mitochondrial diseases.

	Positive aspects	Negative aspects
Apoptosis	The immune system relies on cell apoptosis to eliminate unwanted T and B cells, such as those that target autoantigens in autoimmune diseases. Apoptosis can programmatically remove senescent cells and reproduced cells with extensive genetic errors and cellular damages Xu et al. (2019)	Human immunodeficiency virus (HIV) leads to the apoptosis of T cells in human body, resulting in immune dysfunction Mehrbod et al. (2019)
	Restoring the sensitivity of cancer cells to apoptosis could be used to treat cancer Pistritto et al. (2016)	Excessive apoptosis can lead to a pathological state of the organ, with potentially fatal results Charununtakorn et al. (2016)
Mitophagy	Mitophagy induction may reduce aging-induced cardiovascular damage Ajoolabady et al. (2020)	Excessive mitophagy may lead to cell death, as in pathological conditions of ischaemic stroke Ajoolabady et al. (2020)
	Impaired mitochondrial quality control pathways open up possibilities for treating neurodegenerative diseases Hu et al. (2021)	
NETosis	NETosis, complement system and coagulation system interact and regulate each other de Bont et al. (2019)	NETosis promotes the development of autoimmune diseases Vorobjeva and Chernyak (2020)
	NETosis inhibits pathogens and slows their spread to control infections Vorobjeva and Chernyak (2020)	Excess NET can trigger a cascade of inflammatory responses, exacerbating damage to the lungs, kidneys and other organs Borges et al. (2020)
Pyroptosis	Inducing the pyroptosis of cancer cells is a potential mechanism for cancer treatment Fang et al. (2020)	Pyroptosis plays an important role in the pathogenesis of many cardiovascular diseases Jia et al. (2019)
		Pyroptosis is related to the pathogenesis of many nervous system diseases McKenzie et al. (2020)
Necroptosis	Necroptosis can activates antitumor responses Gong et al. (2019)	RIPK3 is a key mediator of tissue injury in models of acute lung injury (ALI) Siempos et al. (2018), chronic lung diseases Mizumura et al. (2014), and sepsis-associated organ injury Kaiser et al. (2013)
	RIPK3 has anti-viral effects through cell death-independent activities such as promoting the generation of cytokine Nailwal and Chan (2019)	Activation of RIPK3/MLKL–dependent necroptosis happens in I/R-induced AKI model Chen et al. (2018)
	Necrosis plays a key role in promoting the muscle stem cell proliferation and facilitating muscle regeneration Zhou et al. (2020)	RIPK1-mediated inflammatory response is important in chronic inflammation and hepatocellular carcinoma Kondylis and Pasparakis (2019)
		Necroptosis-associated proteins such as PIPK3 and PIPK1 are therapeutic targets for a variety of cardiovascular diseases and neurodegenerative disease Lin et al. (2013); Ofengeim et al. (2015)
		Necroptosis has pro-metastatic and immunosuppressive effects Gong et al. (2019)
Entosis	Entosis can limit the transformed growth of tumor cells cultured in soft agar, and may have a potential role in tumor suppression Overholtzer et al. (2007); Sun et al. (2014)	Entosis leads to the induction of aneuploidy, thus promoting tumor progression Krajcovic et al. (2011)
	Entosis plays physiological role in embryo implantation Li et al. (2015)	
Parthanatos	More research is needed	Parthanatos plays a vital role in Ischemic Stroke Liu et al. (2022)
		Parthanatos is associated with the development of pulmonary hypertension Lv et al. (2021)
		Parthanatos and its related components play a key role in tumor cell proliferation, progression and metastasis Zhou et al. (2021)
		PARP-1 is involved in the pathological process of neurodegenerative diseases by causing mitochondrial dysfunction, regulating gene expression and interacting with a variety of nuclear proteins Wang and Ge (2020)
		Parthanatos contributes to pathogenesis of retinal diseases Greenwald and Pierce (2019)
Ferroptosis	The model system for studying ferroptosis is relatively well developed Stockwell et al. (2017)	Oxidative stress through excess iron is associated with the development of cancer Toyokuni et al. (2017)
	Ferroptosis is associated with other cell death pathways such as apoptosis and necroptosis, thus simultaneous intervention can lead to better results Skouta et al. (2014)	
Autosis	Autosis is involved in the death of pancreatic cancer cells Akimoto et al. (2015); Liao et al. (2016)	Autosis happens in acute liver cell damage in patients with anorexia nervosa Kheloufi et al. (2015)
	Autosis involves in eradicate HIV-infected macrophages and CD4 (+) T cells Zhang et al. (2018); Zhang et al. (2019)	Autosis contribute to ischemia and reperfusion injury Matsui et al. (2007); Liu et al. (2013)
Alkaliptosis	Alkaliptosis is selectively toxic to cancer cells and not to normal cells Song et al. (2018)	More research is needed. The exact upstream mechanism of circadian DRP1 phosphorylation remains to be elucidated Schmitt et al. (2018)
	Alkaliptosis has potential analgesic properties and may therefore be particularly useful for cancer patients with symptoms of pain and anxiety Song et al. (2018)	
Clockophagy	Evidence for the integration of cellular metabolism with the biological clock is consistent, and the molecular mechanisms are becoming clear Ribas-Latre and Eckel-Mahan (2016)	Disturbances in the biological clock may even be a key initiating factor in diseases associated with impaired mitochondrial function, including neurodegenerative diseases such as Alzheimer’s disease Schmitt et al. (2018)
	Circadian regulation of DRP1-dependent mitochondrial structures plays a key role in the control of circadian metabolism and is closely linked to mitochondrial dynamics Westermann (2010)	If mitochondrial dynamics are weakened, then the heart will age faster Song et al. (2017)
Oxeiptosis	Auriculasin can promote colorectal cancer cell oxeiptosis by inducing ROS generation, thus inhibiting cell viability, invasion and clone formation Wang et al. (2022)	Oxeiptosis can protect the body from inflammation induced by ROS or ROS-producing factors such as ozone exposue and viral pathogen Holze et al. (2018); Sokolowska et al. (2019)

## Mitochondria and Cell Death

### Mitochondria and Apoptosis

**FIGURE 2:**
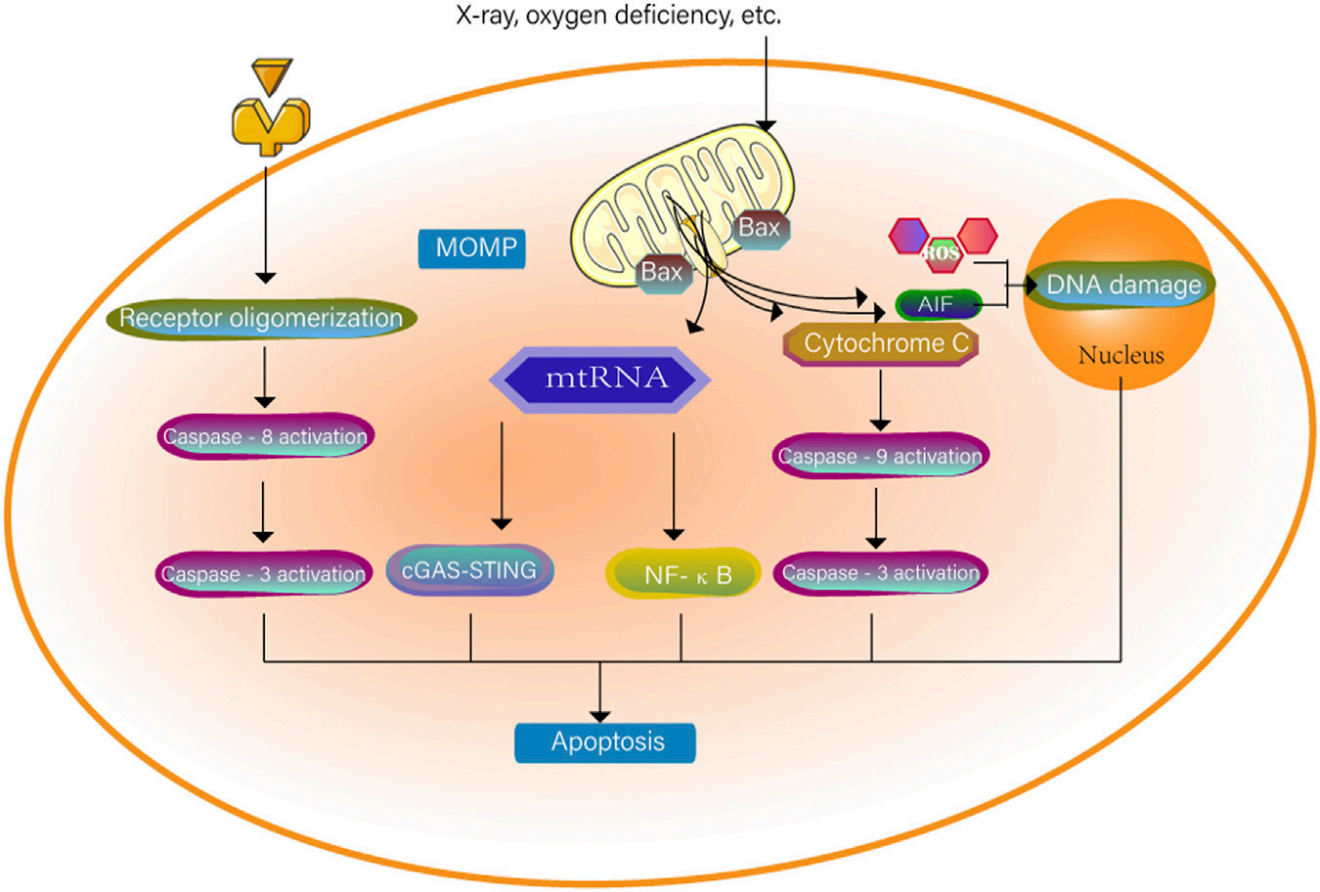
The cell surface death receptor is activated through an exogenous pathway. The cytochrome C of the mitochondria where mitochondria outer membrane permeability occurs enters the cytoplasm. The two pathways together activate caspase-3 to cause apoptosis. mtRNA can also independently cause apoptosis through the NF-κB pathway and the cGAS-STING pathway. When the permeability of mitochondria is increased, the mitochondria will be induced to release apoptosis-inducing factors (AIF). AIF released into the cytoplasm or nucleus will destroy DNA together with ROS and trigger cell apoptosis.

Apoptosis is primarily a caspase-dependent programmed cell death mediated by apoptosomes. Caspase-3 is a key protein commonly found in the pathways of apoptosis and pyroptosis and is a bridge connecting the two modes of death, apoptosis and coking death. Apoptosis consists of endogenous and exogenous pathways. Both internal and external apoptosis signals can activate the caspase protein and initiate programmed death. The specific mechanism is shown in Figure 2. The endogenous pathway is related to mitochondria (Jiang et al., 2020). Mitochondrial contents enter the cytoplasm after mitochondrial integrity is impaired. Cytochrome C is the key signalling molecule that causes apoptosis; it can cause downstream caspase cascade activation (Dunham-Snary et al., 2018), and the Bax/BAK pores mediate the release of cytochrome C and mitochondrial DNA (mtDNA) from the mitochondria (Cosentino and Garcia-Saez, 2018; Riley et al., 2018). mtDNA released into the cytoplasm activates the cGAS-STING pathway, and dying cells induce IFN-β transcription and IFN-β secretion to support caspase-independent cell death. At the same time, mitochondria outer membrane permeability triggers TNF-dependent necrosis in the form of caspase-independent cell death and activates the NF-κB pathway in the absence of caspase (Giampazolias et al., 2017). Increased levels of mitochondrial ROS also induce apoptosis (Wang X. et al., 2017). Abnormally elevated ROS induce cell oxidative stress, destroy cellular structure and mitochondrial outer membrane permeability, and lead to apoptosis (Yuan et al., 2020). Increased mitochondrial permeability is an important factor inducing apoptosis-inducing factor (AIF) release. (AIFs) released into the cytoplasm or nucleus cause DNA damage, leading to apoptosis. ROS are involved in both caspase-dependent and caspase-independent pathways, and thus ROS are an important bridge between these two types of apoptosis (Chen et al., 2020).

In summary, mitochondria can participate in apoptosis either through the caspase-dependent pathway induced by exogenous or endogenous factors or through the caspase-independent pathway induced by mtDNA. Many studies have treated cancers by raising ROS levels to induce apoptosis (Cui et al., 2018).

### Mitochondria and Mitophagy

Mitophagy can maintain the balance between the quality and quantity of mitochondria (Pickles et al., 2018), the survival of cells under starvation and harsh conditions, and the stability of the intracellular environment (Wu and Chen, 2015; Ploumi et al., 2017).

In mitochondrial dynamics, mitochondria achieve efficient oxidative phosphorylation, efficient transport and regulation of mitochondrial autophagy through continuous fusion and fission (Chan, 2020). Mitochondrial fission separates the damaged part from the healthy part of the mitochondria, while mitochondrial fusion allows the combination of two mitochondria to achieve genetic complementation, resulting in a functional mitochondrion (Giacomello et al., 2020). Mitochondrial fission is a multistep process. Contraction and cleavage of the mitochondrial inner membrane is involved through a process mediated by dynamin-related protein 1 (Drp1) and its binding partners. Drp1 is a cytoplasmic protein that lacks a membrane-anchored structural domain and, together with its binding partners, wraps around mitochondria to promote mitochondrial division (Pyakurel et al., 2015). Under normal physiological conditions, mitochondria undergo fission to coordinate the proliferation of epithelial cells, allowing daughter cells to acquire sufficient numbers of mitochondria. However, under pathological conditions, mitochondrial fission may trigger undesirable mitochondrial apoptotic pathways (Ma et al., 2018). Mitofusins (Mfn1/2) and optic atrophy 1 (OPA1) are located on the outer mitochondrial membrane (OMM) and the inner mitochondrial membrane (IMM), respectively. The fusion of the two mitochondria facilitates the mixing of mitochondrial contents, including mitochondrial DNA, metabolites and mitochondrial resident proteins, to remodel structural organization and restore the impaired functional capacity of the mitochondria (Park et al., 2014).

Mitochondrial fragmentation caused by an imbalance in mitochondrial division and fusion is a prerequisite for mitochondrial autophagy. Damaged mitochondrial membrane depolarization can activate PINK-Parkin-mediated mitochondrial autophagy. FUNDC1 is a bridge between mitochondrial fusion fission and autophagy. Through FUNDC1, mitochondrial dynamics and mitochondrial autophagy interact to maintain mitochondrial quality and function (Chen et al., 2016).

**FIGURE 3:**
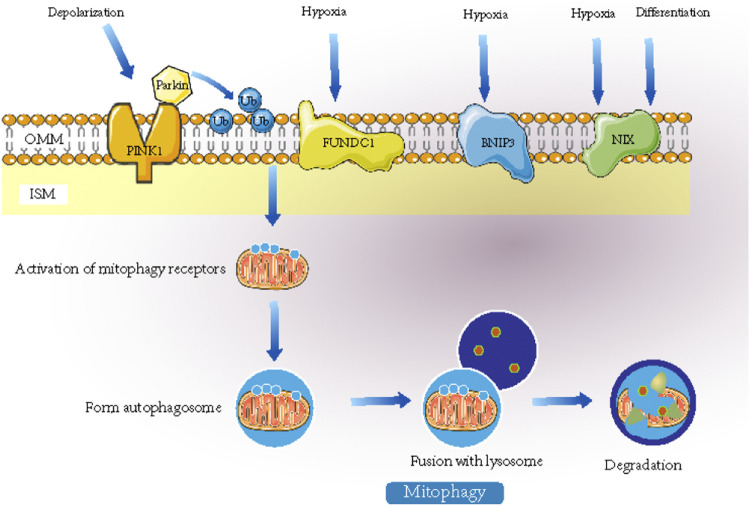
When stimulated by signals such as hypoxia, mitophagy receptors or recruitment of ubiquitin-autophagy adaptors will be activated. They can promote the formation of autophagosome and the fusion with lysosomes and ultimately mediates the degradation of mitochondria. Mitochondrial autophagy mediated by Nix plays an important role in the maturation of mammalian erythrocytes. FUNDC1 can interact with LC3 to mediate mitochondrial autophagy induced by hypoxia.

The mechanisms of mitophagy are divided into two categories, namely, mitochondrial autophagy mediated by the PINK1-Parkin pathway and the receptor-initiated pathway. In the PINK1 (PTEN-induced kinase 1)-induced pathway, Parkin plays a role downstream of mitophagy, and PINK1 regulates the activity of Parkin during mitochondrial depolarization (Onishi et al., 2021). In damaged mitochondria, alterations in mitochondrial membrane potential and PARL protease activity promote the recruitment of PINK1 to the OMM. PINK1-mediated phosphorylation converts Parkin from an inactive to an active form upon mitochondrial depolarization. Parkin then ubiquitinates some OMM proteins, and the polyubiquitinated proteins are recognized by p62 and interact with LC3 (light chain 3) to form autophagic vesicles. The polyubiquitinated proteins target mitochondria and produce phagosomes around mitochondria to remove damaged mitochondria (Narendra et al., 2008; Lin et al., 2019). Overall, PINK1 and Parkin mediate defective mitochondrial clearance (Matsuda et al., 2010). In addition, in the receptor-initiated pathway, FUNDC1 (FUN14 domain-containing protein 1) functions as a receptor for hypoxia-induced mitophagy and is an OMM protein that, when dephosphorylated, interacts with LC3 and induces mitophagy (Zhou H. et al., 2018; Ajoolabady et al., 2020). In adverse conditions such as hypoxia, stimulation of FUNDC1 receptors activates mitophagy receptors, which further promotes the formation of autophagosomes that mediate mitophagy. The mechanisms of mitophagy are shown in Figure 3.

In recent years, there have been some new studies on the mechanism of mitophagy. To clarify the regulatory mechanism of mitochondrial autophagy under oxidative stress, Shu, L. et al. (Guan et al., 2021) screened and identified a series of mitochondrial autophagy regulatory factors. ATAD3B is a new mitochondrial autophagy receptor that mediates mitophagy under oxidative stress and clears damaged mtDNA through this pathway. This protein provides a new target for the treatment of mitochondrial diseases caused by mtDNA mutations. With regard to the negative regulation of mitochondrial autophagy, it is known that USPs (ubiquitin-specific proteases) and the PTEN-L (phosphatase and tensin homologue-long) phosphatase negatively regulate the Parkin pathway (Zhu et al., 2021). Lin et al. (Lin et al., 2020) revealed a new pathway for the regulation of mitochondrial autophagy mediated by PINK1 and its new kinase substrate TUFm. TUFm is traditionally regarded as a nuclear-encoded translation extension factor of mitochondrial proteins. This study showed that PINK1 can transform the mitochondrial autophagy function of TUFm into an inhibitory function by phosphorylating a conservative serine site of TUFm. This kind of signal with bidirectional regulation ability can prevent mitochondria from being excessively cleared and improve the anti-jamming function of the signal regulation loop.

### Mitochondria and NETosis

**FIGURE 4:**
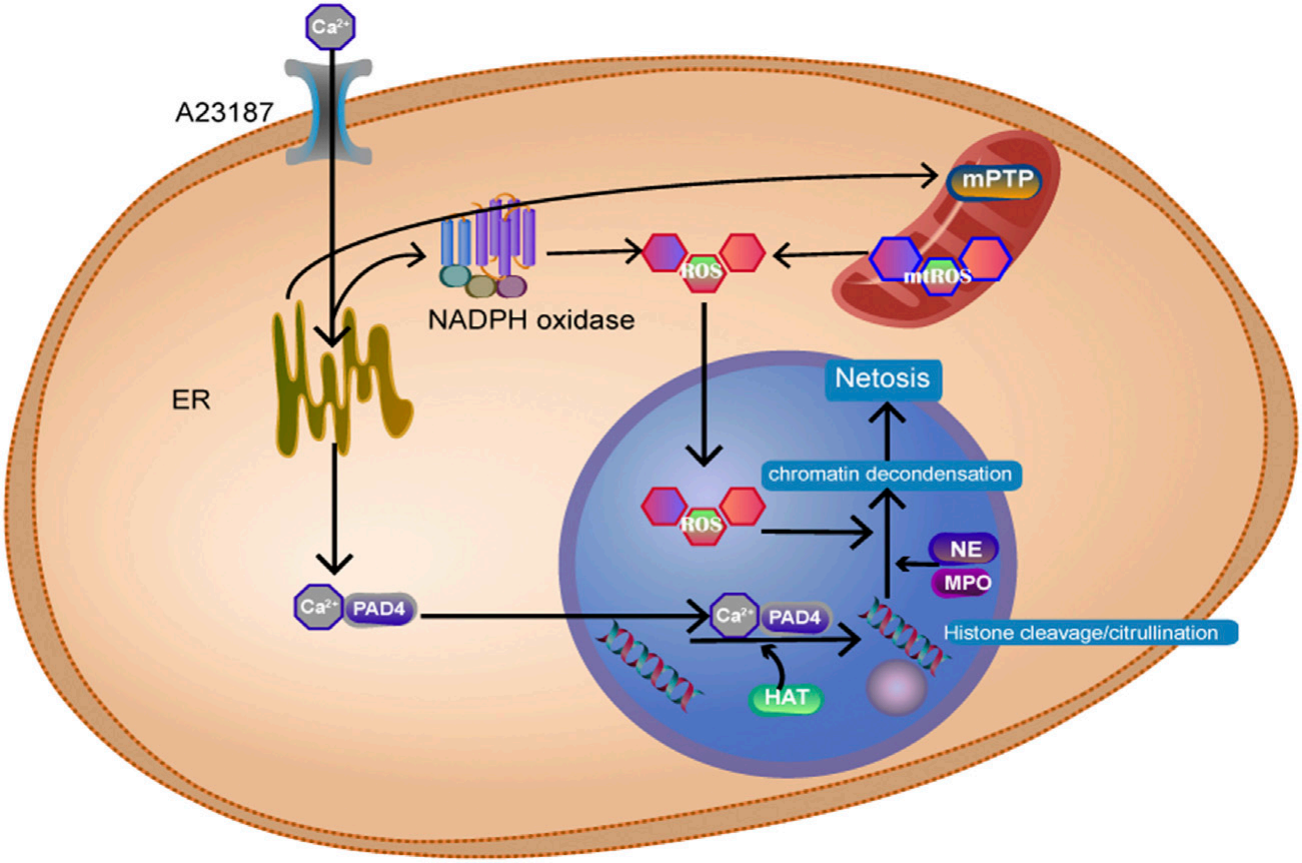
Ca2+ activates NADPH oxidase, produces ROS, and Ca2+ binds to peptidyl arginine deiminase 4 (PAD4+) protein into the nucleus, resulting in histone citrullination. Histone acetyl transferase promotes histone acetylation. Myeloperoxidase (MPO) and neutrophil elastase (NE) enter the nucleus and act on histone, causing chromosomes to dissolve.

NETosis is an inflammatory form of neutrophil death. In this process, the nuclear membrane of the cell ruptures, and chromatin solution binds to intracellular proteins to form cellular capture nets known as neutrophil extracellular traps (NETs). NETs kill pathogens after being released from the cell. Classical NETosis can cause chromosomal depolymerization associated with histone modifications and disruption of the nuclear membrane in pathogens. We can often see the release of granule components into the cytosol under the microscope (Vorobjeva and Chernyak, 2020). The formation of NETs can be divided into the NADPH oxidase (NOX)-dependent pathway and the NOX-independent pathway. The specific mechanism is shown in Figure 4. Takishita et al. (Takishita et al., 2020) showed that extracellular DNA release was significantly lower in mtDNA-deficient neutrophil-like cells than in HL-60 cells after A23187 (a calcium ionophore) stimulation. However, there was no difference in extracellular DNA release induced by phorbol myristate acetate. These results suggest that mitochondrial function is critical to the net formation of the NOX-independent pathway. The team also inhibited NOX-dependent and NOX-independent pathways by inhibiting the generation of mitochondrial reactive oxygen species (mtROS), which reduced the occurrence of NETosis, which showed that mtROS works through both pathways. Therefore, mitochondria can be an alternative source of mtROS even when NOX is lacking (Lood et al., 2016). Mitochondria swell when neutrophils develop NETosis. This suggests that we can predict the occurrence of NETosis by observing mitochondria in advance (Vorobjeva et al., 2020).

NETs can also regulate mitochondrial stability by affecting mitochondrial division, fusion, and autophagy, which has a significant impact on cancer cells (Yazdani et al., 2019). NETosis is involved in many inflammatory diseases and in the development of autoimmune diseases. Low-density granulocytes (LDGs), a unique subpopulation of neutrophils present in individuals with SLE, are characterized by increased NET formation. The Christian Lood team (Lood et al., 2016) demonstrated that ROS inhibitors can significantly inhibit NETosis and lupus-like autoimmune disease in mice.

### Mitochondria and Pyroptosis

**FIGURE 5:**
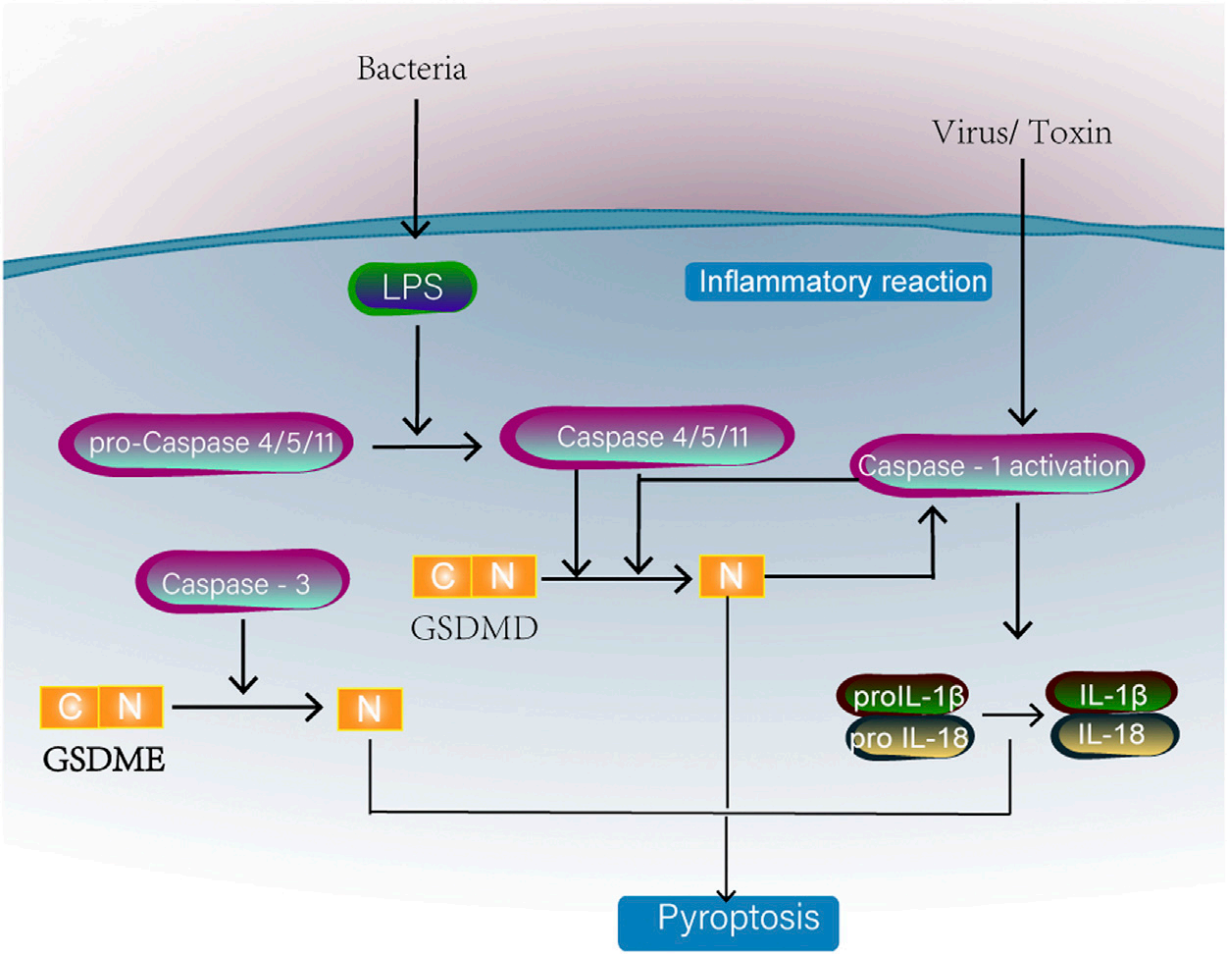
Classical caspase-1 or caspase4/5/11 activation will cleave one of the gasdermin D family proteins. The N-terminal domain (PFD) generated by GSDMD separation oligomerized in the membrane to form large pores, causing membranolysis and cell death. Caspase-1 can also promote the formation of IL-18 and IL-1β. Caspase-3 activation can also induce pyroptosis by cleavage of GSDME.

Pyroptosis is a process of cell death accompanied by the release of a large amount of inflammatory factors. The specific mechanism is shown in Figure 5. Pyroptosis plays an important role in exogenous and endogenous risk signals and is widely involved in the development of diseases such as neurological diseases, atherosclerosis and metabolic diseases. MtROS have long been considered a key signalling molecule for pyroptosis. It further promotes the efficiency of the Gasdermin D cleavage by caspase-1 by oxidizing Gasdermin D (Wang Y. et al., 2019). The NLRP3 inflammasome mediates pyroptosis by activating caspase-1 and then inducing IL-1β and IL-18 maturation. In addition, the activation of NLRP3 requires Ca2+ conduction. Excessive Ca2+ can cause mitochondrial Ca2+ overload damage to mitochondria, stimulating ROS production (Murakami et al., 2012). Sang Hyeon Yeon et al. (Yeon et al., 2017) showed that phospholipid oxidation occurs during cell injury and induces the accumulation of oxidized phosphatidylcholine. It can induce the production of mtROS, which in turn activates the NLRP3 inflammasome. Therefore, Xinyang Yu et al. (Yu et al., 2019) have proposed inhibiting the activation of NLRP3 and pyroptosis by reducing mitochondrial autophagy. Additionally, studies have found that compared to *Porphyromonas gingivalis,* outer membrane vesicles stimulate macrophages to produce cell pyrotopsis signals and induce metabolic shifts from oxidation phosphorylation to glycolytic enzymes. It is thought that this is related to the ability of *Porphyromonas gingivalis* to inhibit ATP and ROS synthesis, and further research is needed to elucidate the underlying reasons (Fleetwood et al., 2017). Currently, many drugs can improve the mitochondrial structure by inhibiting substances that cause mitochondrial damage, including Drp1 (Liu R. et al., 2020; Zou et al., 2020), or directly inhibit the activation of NLRP3 (He et al., 2017). Iron has been shown to be associated with cellular death mechanisms. Iron-activated ROS can induce pyroptopsis through the TOM20-Bax-caspase-gsdme pathway. When iron is used in combination with ROS induction drugs in iron-deficient tumour patients, iron can enhance ROS signal transduction and achieve antitumour effects. This is a very promising treatment for melanoma (Zhou B. et al., 2018).

### Mitochondria and Necroptosis

Cell necrosis is uncontrolled cell death caused by a large extracellular stimulus. These stimuli include physicochemical factors (e.g., calcium overload, oxidative stress, chemicals), biological factors (certain pathogens), and ligand/cytokine effects (Vanlangenakker et al., 2012). It is characterized by cell swelling, shrinking of organelles, disintegration of the plasma membrane, spilling of intracellular DAMPs out of the cell (Kaczmarek et al., 2013), and triggering of an inflammatory response.

**FIGURE 6:**
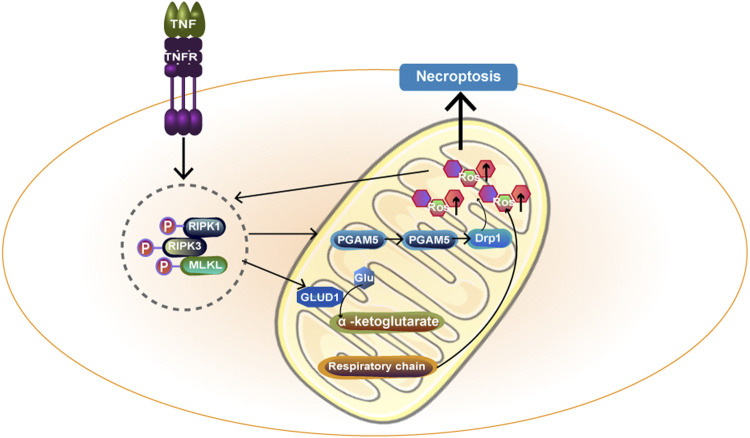
TNF-α acts on cell surface TNFR and transmits death signals through RIPK1 and RIPK3, forming RIPK1/RIPK3/MLKL necrosome. Necrosome activates PGAM5 on the mitochondrial membrane and then PGAM5 enters the cytoplasm, further activating Drp1 and ultimately promoting mtROS formation. In addition, necrosome can act on GLUD1 and catalyze the formation of *a*-ketoglutarate by Glu.α -ketoglutarate acts as an energy substrate to participate in the respiratory chain and promote the generation of mtROS. Ultimately, necroptosis happens. MLKL:mixed gene-domain-like proteins.

In recent years, an increasing number of studies have shown that cell necrosis is also regulated by intracellular molecules, and this new mode of death is known as necroptosis (Degterev et al., 2005). Programmed cell necrosis is primarily initiated by the TNFR and TOLL-like receptor families, followed by the death signal induced by the interaction of protein kinase (RIPK) 1 and receptor-interacting RIPK3, as well as the recruitment and phosphorylation of mixed gene-domain-like proteins (MLKL). Zhao, X et al. (Zhao et al., 2021) found that RIPK3 promotes mitochondrial energy metabolism and mtROS production by upregulating PYGL and PDC-E1 *a*. In addition, RIPK3 can upregulate the level of mitochondrial NOX4 through a posttranscriptional mechanism, resulting in mitochondrial damage. Then, mtROS are upregulated and released extracellularly, which are then recognized as a damage-associated molecular pattern (Uni and Choi, 2021). In addition, mtROS can in turn promote the autophosphorylation of RIPK1 and the recruitment of RIPK3, which are essential for necroptosis (Schenk and Fulda, 2015). Thus, mtROS are involved in signal transduction in programmed cell necrosis. The RIPK1/RIPK3/MLKL necrosome can be transported to mitochondrial membranes and activated by members of the family of phosphate glycerin mutase located in the outer membrane of mitochondria5 (PGAM5). PGAM5 further activates power-related protein 1 (dynamin-protein 1, Drp1), disrupts mitochondrial metabolism and inhibits glutathione, resulting in reduced free radical removal capacity and increased mtROS (Wang et al., 2012). In addition, the RIPK1/RIPK3/MLKL necrosome can also affect metabolic enzymes in the mitochondrial matrix to promote the production of mitochondrial reactive oxygen species (Han et al., 2018). The specific mechanism is shown in Figure 6. However, another study has suggested that the presence of mitochondria is not necessary for necroptosis. Tait SW et al. (Tait et al., 2013) used a method that forced mitochondrial phagocytosis to deplete mitochondria and forced RIPK3 activation by chemically induced dimerization. The results showed that necroptosis is independent of mitochondria and has the same kinetics. mtROS production accompanies but does not lead to RIPK3-dependent necroptosis.

In conclusion, mitochondria are associated with necroptosis, and understanding these mechanisms may be useful in increasing tumour sensitivity to chemotherapy (Han et al., 2018), alleviating kidney disease damage (Uni and Choi, 2021), and serving as therapeutic targets for liver disease (Xue et al., 2020).

### Mitochondria and Entosis

Entosis is a process in which one living cell enters the cytoplasm of another of the same or different type of cell, after which death mainly occurs because in some cases, certain cells can escape this (Overholtzer et al., 2007). Its occurrence may be due to the deficiency of nutrients and growth factors (Florey et al., 2011) and hypoxia (Wang et al., 2016). However, the relationship between mitochondria and entosis has not been clarified. When cells lack oxygen and mitochondria are damaged, entosis can act as a self-repair mechanism to eliminate damaged mitochondria in cells (Wang et al., 2016). In addition, during entosis, the mitochondria of outer cells are redistributed, and the mitochondrial membrane potential of inner cells can be maintained for a period of time, probably due to the energy required by other organelles to maintain function (Garanina et al., 2015). Kohashi, K et al. (Kohashi et al., 2021) found that the mitochondrial membrane potential of scribble (a tumour suppressor protein)/Ras double-mutant cells increased, leading to an increase in mtROS. Inhibition of the mitochondrial membrane potential and reduction in mtROS can reduce the phagocytosis of surrounding scribble-gene knockout cells. This is related to the cannibalism of tumour cells during cancer development. Therefore, we speculated that mitochondrial membrane potential and mitochondrial mtROS production might affect the phagocytosis of nearby cells. In conclusion, the specific molecular mechanism of entosis needs to be further explored to further understand the role of mitochondria in entosis.

### Mitochondria and Parthanatos

**FIGURE 7:**
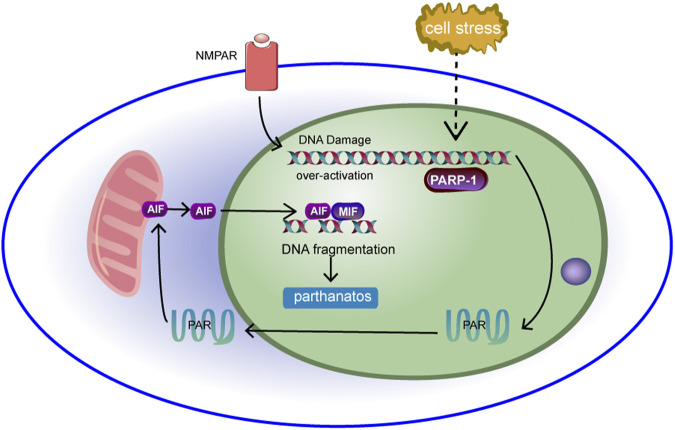
DNA is damaged under cell stress, leading to over-activation of PARP-1. Activation of PARP-1 results in the production of a large number of PAR, which accumulates in the nucleus and transfers from the nucleus to the cytoplasm and into the mitochondria. The binding of PAR to mitochondrial AIF can induce AIF to transfer to the nucleus.Then AIF interacts with macrophage migration inhibitor (MIF), causing chromatin to agglutinate and leading to cell line death.

Parthanatos is a poly (ADP-ribose) polymerase-1 (PARP-1)-dependent cell death that occurs independently of caspases (Fatokun et al., 2014; Galluzzi et al., 2018). PARP-1 is excessively activated and leads to parthanatos in pathological conditions (Andrabi et al., 2008). In the nucleus, PARP-1 catalyses the synthesis of poly (ADP-ribose) (PAR), a polysaccharide. Therefore, PAR increases with the activation of PARP-1. PAR acts as a cell death signal, inducing the release of apoptosis-inducing factor (AIF) and its migration from mitochondria to the nucleus (Yu et al., 2006). However, the specific mechanism by which PAR induces AIF release remains unclear. Some scholars believe that the release of AIF may be related to calpain and Bax. Moubarak, R. S. et al. (Moubarak et al., 2007) showed that after activation of PARP-1, exonuclear calpain was activated, and calpain further activated Bax, a member of the proapoptotic B cell lymphoma/leukaemia-2 (Bcl-2) family. At this time, AIF in mitochondria was activated and released from mitochondria under the action of Bax. In addition, studies by Vosler, P. S. et al. (Duan et al., 2007) showed that N-methyl-d-aspartate (NMDA) activation of the NMDA receptor induces mitochondrial Ca2+ disorder. Excess Ca2+ leads to mitochondrial release of O2·−, which reacts with NO to generate ONOO-, which further leads to DNA damage. PARP-1 is then further activated, resulting in parthanatos. Excess Ca2+ also induces calpain overactivation, which further mediates the formation and release of mature AIF (Qiu et al., 2020). In the nucleus, the enhanced ability of deubiquitinated AIF to bind to DNA (Luo et al., 2021) causes large-scale DNA disruption and chromatin condensation. This is considered to be the cause of cell death (Fatokun et al., 2014). The specific molecular mechanism of parthanatos in mitochondria is shown in Figure 7.

In conclusion, parthanatos is associated with a variety of diseases, especially neurological diseases, such as various neurodegenerative diseases (Fan et al., 2017). Therefore, regulating the activity and function of PARP-1, PAR and AIF to regulate parthanatos may play a positive role in disease treatment.

### Mitochondria and Ferroptosis

**FIGURE 8:**
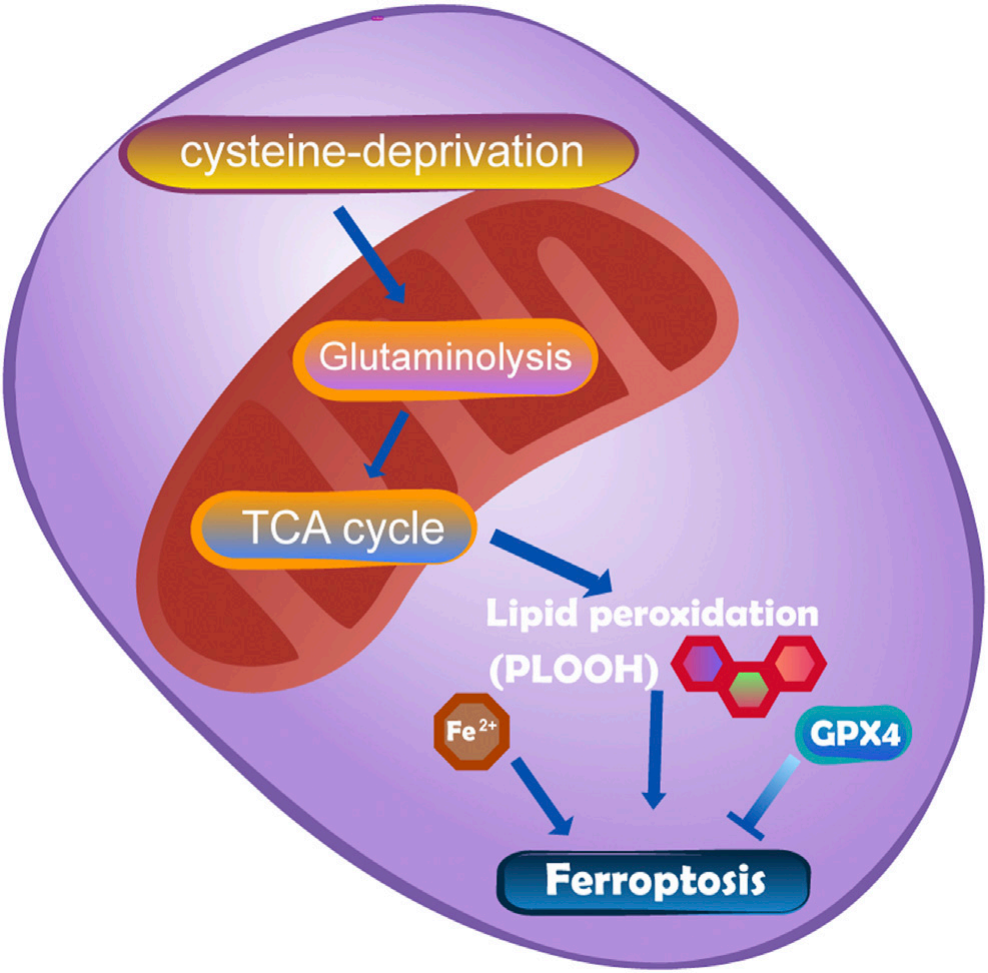
The mutation of fumarate hydratase (FH) can resist CDI ferroptosis by inhibiting TCA cycle and electron transport chain.DHODH works in parallel with mitochondrial GPX4 to inhibit ferroptosis in the inner membrane of mitochondria by reducing ubiquinone to panthenol.

Ferroptosis was discovered in 2003 when Dolma et al. researched the mechanism of the small molecule erastin in killing tumour cells with mutations in the oncogene RAS (Dolma et al., 2003). Ferroptosis is initiated by two main pathways: exogenous or transporter-dependent pathways (such as system Xc-) and endogenous or enzyme-regulated pathways (such as GPX4) (Gao and Jiang, 2018). In addition, FSP1 has been proposed to be another important pathway mediating ferroptosis. The inhibition of ferroptosis by FSP1 occurs *via* NAD (P)H-catalysed regeneration of ubiquinone (CoQ 10). Gao et al. (Gao et al., 2019) showed that mitochondria play an important role in ferroptosis induced by cysteine deprivation and have nothing to do with ferroptosis induced by GPX4 inhibition. This means that the mechanism of mitochondrial-mediated ferroptosis is different from the pathways described above. The mechanism is that the accumulation of ROS in cells exceeds the redox content maintained by GSH and GSH-based phospholipid hydrogen peroxide enzymes (Ingold et al., 2018). The lack of cysteine can induce the decomposition of glutamine, which drives the hyperpolarization of mitochondrial membrane potential (MMP) and the accumulation of lipid peroxides by decomposing and replenishing the intermediates of the TCA cycle (Gao et al., 2019). The TCA cycle and electron transport chain are the main sources of lipid peroxides produced by cells. The specific molecular mechanism of ferroptosis in mitochondria and cysteine deprivation-induced ferroptosis is shown in Figure 8.

It should be emphasized that the mechanisms of recombinant glutathione peroxidase (GPX4) in the cytoplasm and mitochondria, recombinant dihydroorotate dehydrogenase in mitochondria and FSP1 on the cell membrane are parallel and independent and work in combination defend against ferroptosis. These factors constitute the main defence system in mitochondria. Unlike the previous belief that GXP4 is a necessary target for ferroptosis interventions, the proposed pathways provide new ideas for ferroptosis-based interventions. Ferroptosis plays a dual role in promoting and inhibiting tumours in the process of tumorigenesis. Clarifying the relationship between mitochondria and ferroptosis can provide new ideas for tumour treatment (Stockwell et al., 2017). However, it is still controversial whether mitochondria are involved in the process of ferroptosis. In experiments with Dixon et al., mitochondrial DNA was depleted (R0). Compared with parents, there was no significant difference in sensitivity to iron prolapse (Dixon et al., 2012). In addition, a study on the mechanism of ferroptosis inhibitor cohorts failed to determine the relationship between the mitochondrial localization of ferroptosis inhibitors and their anti-ferroptosis efficacy (Gaschler et al., 2018). Therefore, further studies are needed to explore the relationship between iron death and mitochondria.

### Mitochondria and Autosis

Liu et al. (Liu et al., 2013) characterized a novel autophagy-dependent cell death known as autosis. Autosis is a form of cell death regulated by Na^+^, K^+^—ATPase that has unique morphological characteristics, mainly including an increase in autophagosomes and autolysosomes and nuclear folding (early-mid stages) and focal ballooning of the perinuclear space (late stages). Treatment with autophagy-inducing peptides such as tAT-beclin1 or Tat-vflip α2, starvation, hypoxia-ischaemia and reperfusion can induce autosis. Cardiac glycosides, a type of Na^+^, K^+^—ATPase inhibitor, such as digoxin strophanthidin, digitoxigenin and neriifoli, can save autophagy-inducing peptide and starvation-induced autosis (Liu et al., 2013). In addition, autotic death of the hippocampus after carotid artery ligation in newborn rats can be blocked, and the cerebral infarction area can be reduced by cardiac glycosides (Liu et al., 2013). Current studies suggest that autosis is caused by the interaction between Na^+^, K^+^—ATPase and beclin1. Cardioside destroys the interaction between Na^+^, K^+^ —ATPase and beclin1 by interacting with Na^+^, K^+^—ATPase (Fernández et al., 2020). In addition, interactions between Na^+^, K^+^—ATPase and beclin1 occur in many endoplasmic membranes, including the endoplasmic reticulum, perinuclear membrane, endosome and mitochondria (Nah et al., 2020). Therefore, Na^+^, K^+^—ATPase/beclin1 interactions may trigger autosis through their effects on mitochondrial function. At present, the role of mitochondria in autosis needs to be further explored.

### Mitochondria and Alkaliptosis

**FIGURE 9:**
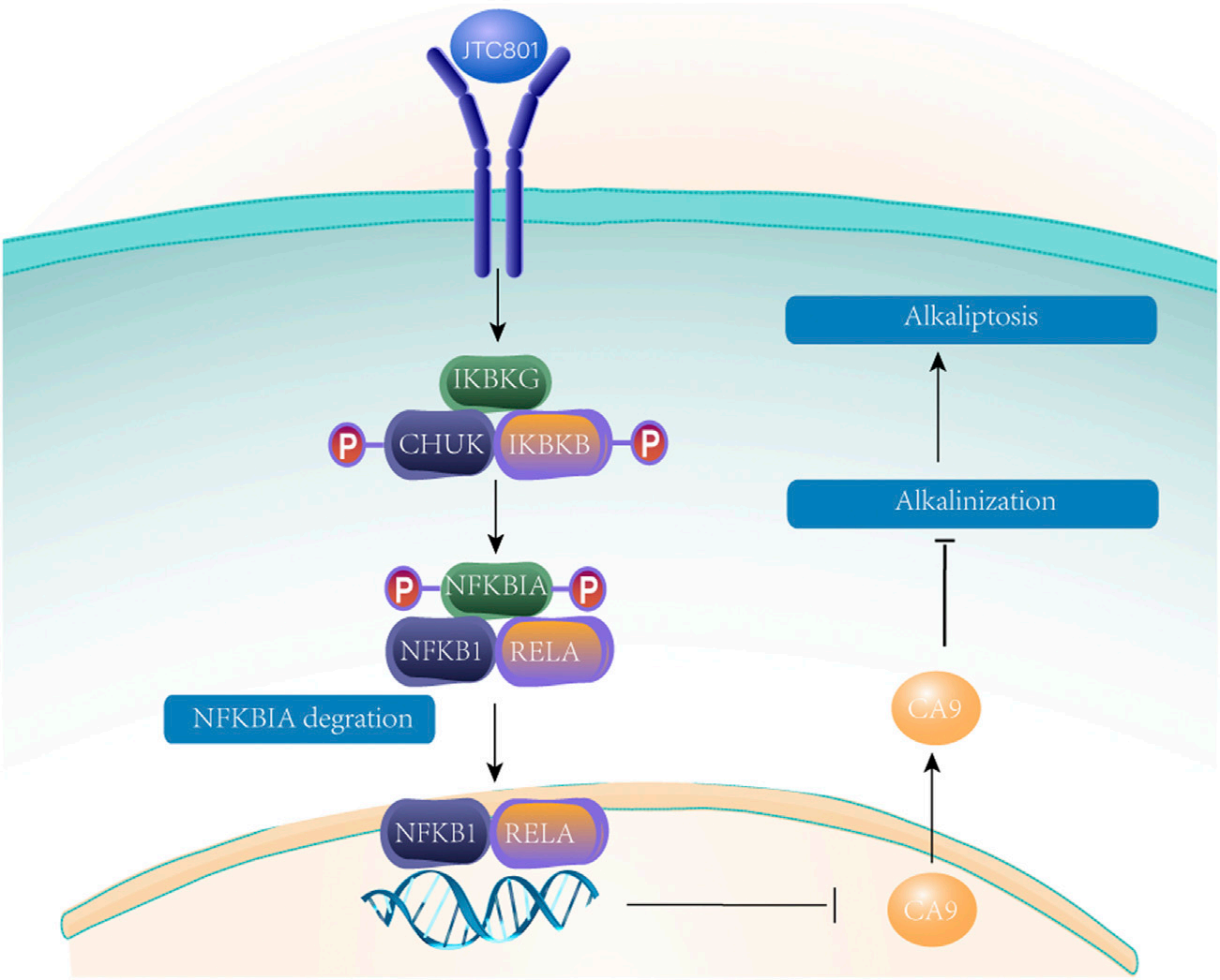
After JTC 801 recruits and activates the IKK protein complex, the IKK protein complex phosphorylates and degrades NFKB inhibitor *a* (NFKBIA). Nuclear factor kappaB subunit 1 (NFKB1) or RELA occurs nuclear translocation, which regulates gene expression and down-regulates CA9 blocking NF- *κ* B pathway.

Alkaliptosis is a new type of RCD caused by intracellular alkalization (Song et al., 2018). Yamada H et al. (Yamada et al., 2002) identified JTC801 when screening for cytotoxicity against pancreatic cancer cell lines to identify cytotoxic drugs. JTC801 has been shown to be a selective antagonist of opioid-associated nociceptin receptor 1 (OPRL1). Opioid-associated nociceptin receptor 1 is a nociceptin receptor distributed throughout the brain. Alkaliptosis can effectively kill a variety of cancer cells, and the cytotoxicity is different from any previous RCD mode. Oxidative stress and ion channel activation are not necessary in JTC801-induced alkaliptosis (Song et al., 2018). Experiments show that JTC801-induced cell death can be blocked only by inhibiting intracellular alkalinization (Song et al., 2018). Alkaliptosis requires kappa B kinase (IKK) and NF-κB pathway-dependent downregulation of carbonic anhydrase 9. The specific mechanism is shown in Figure 9. Nuclear factor kappa B subunit 1 (NFKB1) or RELA (a subunit of NF-κB that is primarily responsible for its transactivation function.) undergo nuclear translocation, which regulates gene expression and downregulates CA9, blocking the NF-κB pathway. Inhibition of IKBKB (kappa B kinase subunit B) or RELA by using drugs or controlling gene expression can reduce JTC801 to effectively kill human pancreas, kidney, prostate, skin and brain cancer cells through alkaliptosis (Zheng et al., 2018).

Cancer cells can escape apoptosis and invalidate treatment (Brown and Attardi, 2005). Therefore, new and effective anticancer drugs are needed to treat cancer. JTC801 has selective toxicity to cancer cells and no selective toxicity to normal cells. One possible mechanism for this difference is that pH disorder is a common feature of cancer cells. Therefore, we can speculate that the regulation of intracellular pH may be a reasonable method for the treatment of cancer (Webb et al., 2011; Benej et al., 2014). Understanding the acid-base homeostasis of normal cells and cancer cells, as well as information on gene expression patterns under alkalization, may provide insights into the interpretation of phenotypes and the direction of further research. In targeted cancer therapy, the molecular target of JTC801 anticancer activity needs to be further studied.

### Mitochondria and Clockophagy

**FIGURE 10:**
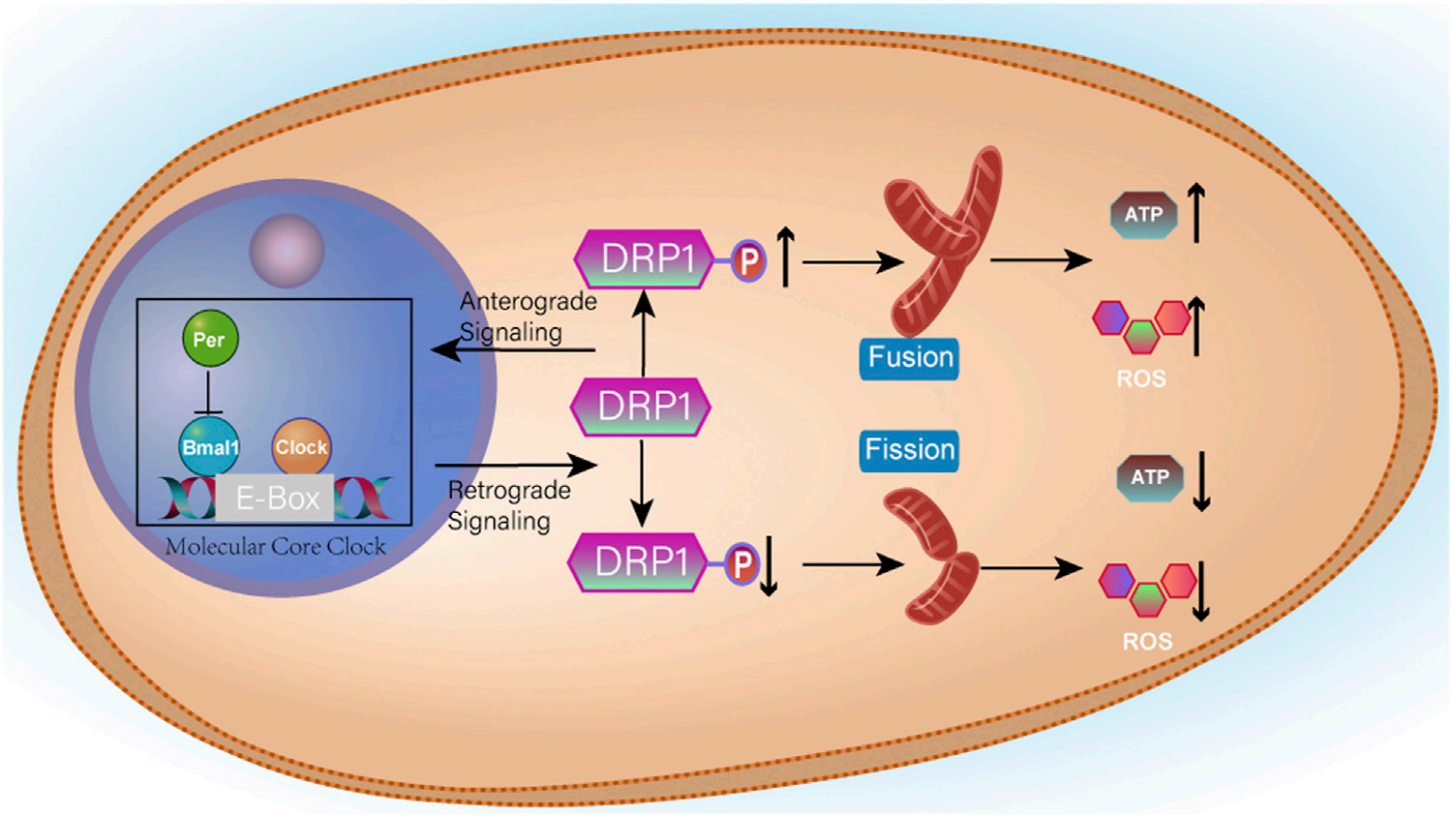
Clock/Bmal1 binds to the E-Box in the promoter of the target gene and activates transcription, whereas Per can inhibit the action of Bmal1. When Drp1 expression is increased, mitochondria fuse and therefore produce more ATP and more ROS. When Drp1 expression is increased, it promotes Per aggregation and inhibits the initiation of transcription, thereby reducing Drp1 expression.

Clockophagy is a hierarchical network of oscillators. There is a central oscillator in the brain, and peripheral oscillators exist in nearly all cells of the body (Mohawk et al., 2012). Circadian rhythms control the metabolism of organisms. Schmitt, K. et al. (Schmitt et al., 2018) showed that clockophagy controls the number of mitochondria by regulating the division and fusion of mitochondria, thus controlling the energy metabolism of cells. The division-fusion cycle of mitochondria is related to the fission protein Drp1. The activator Bmal1 and repressors PER1 and PER2, which are closely related to the biological clock, regulate the gene expression of Drp1 in the core cycle. The division-fusion cycle of mitochondria is related to the fission protein Drp1. If Drp1 fission protein is damaged by drug or genetic means, it will in turn affect the rhythm of the circadian clock. There is a negative feedback mechanism regulating the expression of Drp1 (Shostak, 2017). Moreover, clockophagy is linked to mitochondrial energy metabolism. When clockophagy is disrupted, overall cell productivity is reduced. There is a negative feedback mechanism regulating Drp1 expression. The specific mechanism is shown in Figure 10 (Leong, 2018). This finding provides a new idea for the treatment of mitochondrial dysfunction caused by clockophagy interference.

The relationship between clockophagy, metabolism and lifespan is very complex. Ulgherait, M. et al. revealed that male *Drosophila melanogaster* that lose certain clockophagy components such as PER and Timeless (TIM) can significantly prolong their lifespan as the intestinal mitochondrial uncoupling regulated by clockophagy can prolong the lifespan of *Drosophila melanogaster* and inhibit the excessive proliferation of intestinal stem cells caused by ageing and even tumorigenesis (Giebultowicz, 2018; Ulgherait et al., 2020). This provides a new potential antiageing target.

### Mitochondria and Oxeiptosis

**FIGURE 11:**
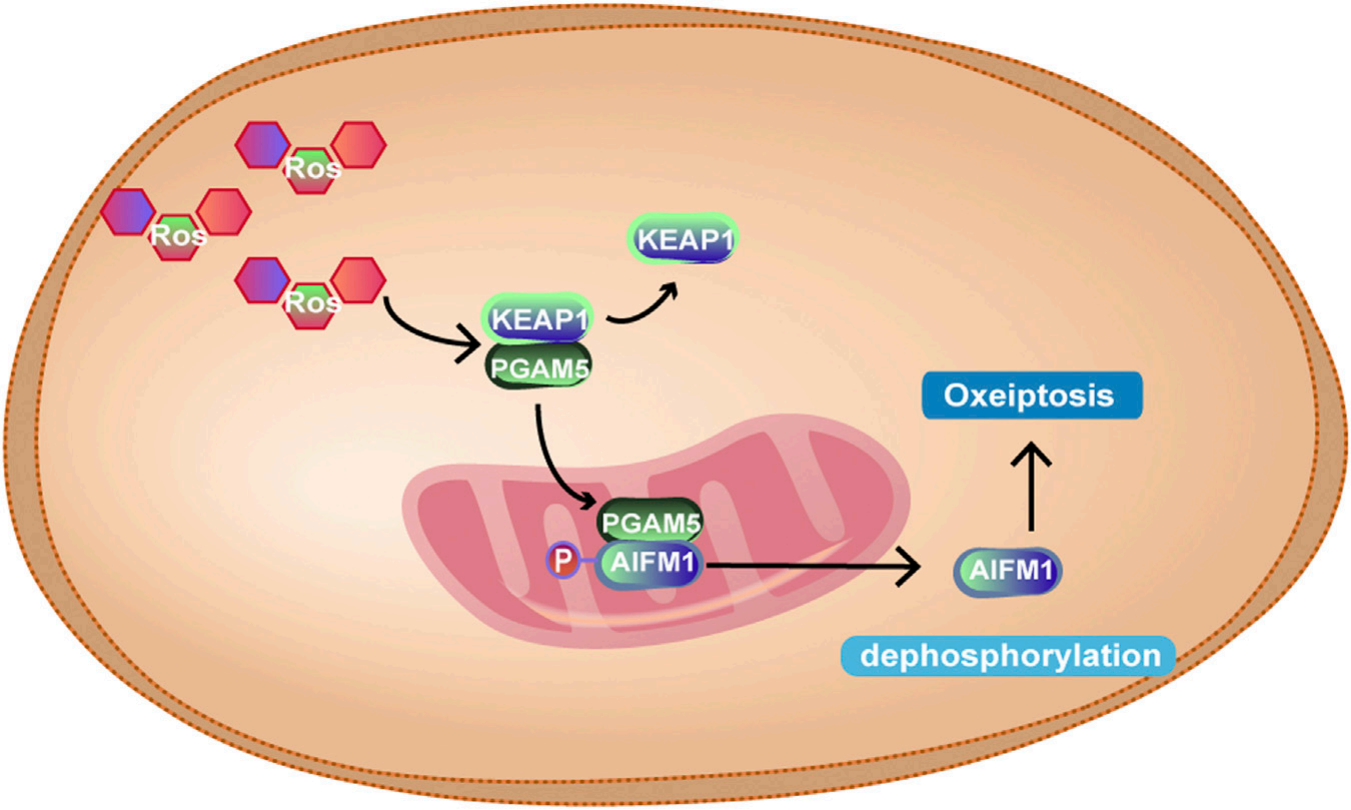
At high intracellular ROS levels, KEAP1 and PGAM5 are separated, and PGAM5 enters mitochondria, binds to AIFM1 and dephosphorylates AIFM1 at Ser116, leading to oxeiptosis. However, the mechanism of oxeiptosis induced by dephosphorylation of AIFM1 is not yet clear.

ROS include superoxide anion free radicals (O2-) and hydroxyl free radicals (OH-). Mitochondrial phosphate oxidation produces trace amounts of ROS that can be eliminated by a variety of antioxidant enzymes in the body. In 2018, Holze et al. (Holze et al., 2018) found that O_3_ or H_2_O_2_ at high concentrations can induce apoptosis-like death, namely, oxeiptosis. Oxeiptosis is a nonmitochondrial-mediated, noninflammatory and ROS-sensitive apoptosis pathway of cell death, which depends on a new signalling pathway, KEAP1-PGAM5-AIFM1. KEAP1 is an ROS sensor that binds Nrf2 in the cytoplasm under physiological conditions. At lower ROS concentrations, KEAP1 is oxidized by ROS, and its conformation changes, which separates from Nrf2 and binds to PGAM5. At this point, Nrf2 enters the nucleus (Scaturro and Pichlmair, 2018) and promotes the transcription of antioxidant genes, such as glutathione peroxidase (GPX) and peroxidase (PRX) (Tu et al., 2019). When excess ROS are produced, PGAM5 is separated from KEAP1 and transferred to mitochondria, where PGAM5 dephosphorylates the Ser116 residues of AIFM1, thus mediating the occurrence of oxeiptosis. As for AIFM1, earlier studies have shown that AIFM1 can translocate into the nucleus, mediating the fragmentation of chromatin (Susin et al., 1999). However, some scholars believe that AIFM1 does not leave the mitochondria but is repositioned as a circular structure in the mitochondria (Scaturro and Pichlmair, 2019). Therefore, the role of AIFM1 in oxeiptosis needs to be further elucidated. The unique signal transduction ability of KEAP1 may be regulated by a large number of cysteine residues at its C-terminus, which may account for its perception and different responses to different ROS levels. Scaturro, P (Scaturro and Pichlmair, 2019) speculated that in response to high concentrations of ROS, a group of alternative residues are oxidized, enabling individual proteins to perform molecular transformations between performing cell protective functions or mediating cell death. How the apoptosis-inducing factor mitochondrion-associated 1 dephosphorylation mediates oxeiptosis needs to be further explored. In addition, the interaction between oxeiptosis and other cell death signalling pathways needs to be further studied. The specific mechanism is shown in Figure 11.

Regulation of ROS-mediated oxeiptosis is expected to be a potential therapeutic target for a variety of diseases. Lack of oxeiptosis may affect the adaptive regulation of ROS. When excessive oxeiptosis occurs, the body may be damaged. Mitochondria, as an important source of ROS and part of the site of oxeiptosis, may be involved in the regulation of oxeiptosis, but the correlation between the two is lacking.

## Mitochondrial and Systemic Diseases

### Mitochondria and Nervous System Diseases

Mitochondrial dysfunction can cause a variety of nervous system diseases (Moreira et al., 2010). At present, the pathogenesis of Parkinson’s disease (PD) is unclear. However, it is clear that PD is associated with mitochondrial dysfunction. The surge in mitochondria is an early feature of PD (Mor et al., 2020). Mor et al. (Mor et al., 2020) found that BCAT-1 (RNAi) knockdown can overactivate nerve cell mitochondria and induce oxidative damage, which can reproduce PD-like symptoms. Ray et al. (Ray et al., 2014) added that exposure to metabolites in neurons expressing human *a*-syn or LRRK2 G2019S disrupts the normal function of mitochondria. In conclusion, much evidence indicates that mitochondrial oxidative stress is a potential cause of PD (Snow et al., 2010). At present, many drugs improve the neurodegeneration of PD by inhibiting mitochondrial oxidative stress and inhibiting nerve cell apoptosis (Pointer and Klegeris, 2017; Liu L. et al., 2020; Johnson et al., 2020). Experiments by David C Schöndorf et al. (Schondorf et al., 2018) showed that the NAD + precursor nicotinamide riboside (NR) can also significantly improve mitochondrial function by increasing mitochondrial autophagy.Targeting mitochondrial Pink1-Parkin-dependent autophagy is a potential therapeutic approach for Alzheimer’s disease (AD). Amyloid *ß* can destroy Parkin and PINK1 in the mitochondrial autophagy pathway, resulting in abnormal mitochondrial autophagy (Manczak et al., 2018). In a primary mouse hippocampal neuron (HT22) model, the accumulation of mAPP and Aβ in the hippocampus causes mitochondrial defects, including abnormal mitochondrial autophagy (Reddy et al., 2018). Drugs developed based on Drp1 show great potential (Manczak et al., 2016). The partial reduction in Drp1 reduces the production of phosphorylated Tau to increase synaptic activity (Kandimalla et al., 2016). In addition, many drugs reduce the level of mtROS, reduce the deposition of Aβ in the brain and improve the function of the central cholinergic system (Li et al., 2017; Zhang et al., 2018b; Hu et al., 2018; Wang C. et al., 2019; Park et al., 2020).Exogenous mitochondrial transplantation has been proved to be effective in the treatment of central nervous system injury in many clinical trials (Nakamura et al., 2020).

In conclusion, neuronal homeostasis is largely dependent on mitochondria. Mitochondrial dysfunction can lead to problems such as impaired energy metabolism, increased ROS production, and neuronal degeneration. Mitochondria affect the release of neurotransmitters from synapses by regulating calcium concentration and energy metabolism. Many studies have linked mitochondrial dysfunction during aging to a variety of neurodegenerative diseases and memory loss (Todorova and Blokland, 2017). Therefore, targeting mitochondrial quality control (MQC) is an important method for the treatment of most neurodegenerative diseases (Hu et al., 2021).

### Mitochondria and Cardiovascular Diseases

At present, there is an increasing number of studies on the role of pyroptosis and cardiovascular diseases worldwide. Among them, the most classic is atherosclerosis. NLRP3 plays an important role in the formation of atherosclerosis (Duewell et al., 2010). When cells take in excessive calcium ions or mitochondrial barriers are damaged, NLRP3 is activated and then aggregates at the mitochondria to form NLRP3 oligomers and activate the caspase cascade. Xianxian Wu et al. (Wu et al., 2018) proved that nicotine induces atherosclerosis by stimulating the production of ROS to activate the classic pyroptosis pathway of caspase-1-dependent endothelial cells. In addition to nicotine, hyperlipidaemia, proinflammatory factors and many other factors are all induced by activating the caspase-1-sirtuin1-activator protein-1 pathway (Yin et al., 2015; Oh et al., 2020). This also suggests that we can treat certain cardiovascular diseases by preventing the activation of caspase-1. Monocytes/macrophages also play different roles in different periods of atherosclerosis. Oxidation of low lipoprotein (OX-LDL) activates NLRP3 in the plaque necrosis area formed early in atherosclerosis (Duewell et al., 2010). Sin Jee Son et al. (Son et al., 2013) demonstrated that TG can also stimulate macrophages to undergo pyrotopsis.

In addition, studies have found that AMP-activated protein kinase (AMPK), which regulates cellular energy metabolism and oxidative stress, can maintain mitochondrial homeostasis by regulating ROS levels in cells and calcium ion levels, thus participating in cardiovascular diseases (Wu and Zou, 2020). Additionally, studies have begun to pay attention to microRNAs in mitochondria. Hüseyin Altuğ Çakmak al. (Cakmak and Demir, 2020). reported on the multiple roles of mitochondrial microRNAs in cardiovascular diseases, including angiogenesis, cardiac cell growth and plaque formation. Currently, related drugs have been developed. Karnewar S et al. (Karnewar et al., 2016) synthesized a new drug, esculin (Mito-ESC), which produces NO through AMP-activated protein kinase and regulates mitochondrial oxidative stress to treat atherosclerosis. Drugs targeting microRNAs also exhibit great potential in the treatment of cardiovascular diseases (Hang et al., 2017). Endothelial cells play an important role in the pathogenesis of various cardiovascular diseases. Mitochondrial dysfunction is an important feature of endothelial injury. Xing Chang et al. proposed targeted therapy of mitochondrial quality control (MQC) to repair microvascular injury and improve myocardial infarction (MI). However, no drugs targeting mitochondrial quality control (MQC) have been proven to treat myocardial infarction (MI) (Chang et al., 2021). Mitophagy is closely related to cardiovascular ageing, and ageing disrupts a variety of mitochondrial functions. Therefore, mitophagy is a promising target for the treatment of cardiovascular ageing (Ajoolabady et al., 2020).

### Mitochondria and Digestive System Diseases

Numerous studies have also shown that targeting PINK1-Parkin-mediated mitochondrial autophagy can also reduce liver damage. Yong Zhang et al. (Zhang et al., 2018a) demonstrated in ethanol-treated HepG2 cells and animal models that gastrodin can inhibit hepatocyte apoptosis by maintaining mitochondrial homeostasis and inhibiting the activation of caspase-1. Moreover, liver physiology was significantly improved in alcoholic liver disease (ALD) model mice when ALD rats were injected with gastrodin at different doses. For nonalcoholic fatty liver disease (NAFLD), Ariel E Feldstein et al. (Feldstein et al., 2003) emphasized the increase in caspase-3/7 activity in patients with nonalcoholic steatohepatitis (NASH), as well as a significant increase in hepatocyte apoptosis and FAS receptors. This suggests the involvement of cell death modes, including apoptosis, in nonalcoholic fatty liver disease. At present, many studies have developed antiapoptotic drugs to treat NASH (Wang P.-X. et al., 2017; Ezquerro et al., 2019; Nasiri-Ansari et al., 2021). Satoshi Tanaka al. (Tanaka et al., 2016). studied Rubicon, which is a negative regulator of autophagosome-lysosome fusion that interacts with beclin1. It is highly expressed in patients with NASH and can accelerate liver cell apoptosis and inhibit autonomy. The author also emphasized that Rubicon can be used as a target to inhibit its expression to treat NASH.

In addition, a variety of enzymes in the mitochondrial electron transport chain can be used as targets for inhibiting mitochondrial function and treating NAFLD. Some studies have also found that inhibition of methylation-controlled J protein (MCJ) can regulate the endogenous respiratory chain complex to reduce ROS production. Inhibition of methylation-controlled J protein has been shown to improve liver cell damage and liver fibrosis, and it is now an effective alternative therapy for NAFLD (Barbier-Torres et al., 2020). Fernandez-Tussy et al. (Fernandez-Tussy et al., 2019) used the activity of the complex IIGNMT in the mitochondrial electron transport chain to reduce fatty acid *ß*-oxidation to treat NASH.

In inflammatory bowel diseases (IBD), inflammation-related mitochondrial dysfunction in the intestinal epithelium leads to metabolic imbalances, resulting in reduced and dysfunctional Paneth cells (Khaloian et al., 2020). Also, Phb1 is a major mitochondrial intima protein that is essential for the function of the mitochondrial respiratory chain. In IBD, its expression is down-regulated, and the loss of intestinal epithelial Phb1 leads to spontaneous ileitis (Jackson et al., 2020).

### Mitochondria and Respiratory Diseases

Mitochondria are associated with many respiratory diseases. Ana L Mora et al. (Mora et al., 2017) linked ageing with pulmonary fibrosis, decreased the expression of the mitochondrial homeostasis regulator mitophagy protein PINK1 in senescent cells, and mitochondrial dysfunction of type II alveolar epithelial cells (AECII), which affected the oxidative stress function of AECII mitochondria and caused lung damage and susceptibility to subsequent fibrosis increases. There are a variety of drugs that target ROS for respiratory diseases. Jennifer L Larson-Casey et al. (Larson-Casey et al., 2016) showed that Akt1 can promote mitochondrial autophagy by increasing the level of ROS to improve the anti-apoptotic ability of lung macrophages and affect the development of pulmonary fibrosis. PFD, as an anti-IPF drug, regulates PDGFR-PI3K-Akt signal transduction by inhibiting the production of mitochondrial ROS, promoting mitochondrial autophagy and reducing the symptoms of pulmonary fibrosis (Kurita et al., 2017). Kenji Kobayashi et al. (Kobayashi et al., 2016) used a mitophagy protein PARK2 knockout mouse model and found that mitochondrial autophagy was inhibited. PARK2 expression reduces mitochondrial autophagy-mediated PDGFR-PI3K-AKT activation, which inhibits the differentiation and proliferation of lung fibroblasts. Jun Araya et al. (Araya et al., 2019) established a mitophagy protein PRKN knockout (KO) mouse model and found that PRKN can eliminate excessive ROS and mitochondrial dysfunction caused by insufficient mitophagy protein PINK1 expression. In contrast, mitophagy protein PINK1 cannot eliminate the effects of PRKN, which indicates that mitophagy protein PRKN plays a key role in regulating mitochondrial autophagy and the pathogenesis of chronic obstructive pulmonary disease (COPD). Saburo Ito et al. (Ito et al., 2015) also found that the reduction in mitophagy protein PRKN levels in COPD patients leads to insufficient mitochondrial autophagy, which is an important mechanism of COPD. Mitochondrial autophagy is closely related to the occurrence of IPF and COPD. COPD is closely related to mitochondrial oxidative stress (Wiegman et al., 2015). NRF2 is mainly expressed in epithelial and alveolar macrophages. It can reduce cellular oxidative stress and has great potential in the treatment of COPD (Boutten et al., 2011; Manczak et al., 2016). Also, in mice lacking sirtuin 3 (a mitochondrial deacetylase), mitochondrial acetylation is increased and many mitochondrial enzymes and complexes are inhibited, thus inhibiting mitochondrial function and leading to the emergence of spontaneous pulmonary arterial hypertension (PAH) (Paulin et al., 2014). As a result, drugs that regulate mitochondrial metabolism may be effective in alleviating PAH. For example, cannabidiol can restore mitochondrial energy metabolism and reduce lactic acid production and glycolysis abnormalities, thus alleviating PAH (Lu et al., 2021).

### Mitochondria and Endocrine Diseases

Mitochondria participate in the secretion of 61 hormones (Chow et al., 2017). In terms of biological mechanisms, mitochondria affect metabolic function in four aspects: bioenergetics, biogenetic and kinetic changes, and excessive production of ROS (Petersen et al., 2003; Houstis et al., 2006; Anderson et al., 2009; Jheng et al., 2012).

Diabetic endocrine diseases are the most common endocrine disorder in patients with hereditary mitochondrial diseases, which can be caused by mitochondrial genome and nuclear gene defects (Chow et al., 2017). The former contains maternally inherited diabetes and deafness caused by the most common mtDNA point mutation in MT-TL119 (Maassen et al., 2004). MT-TL119 is the mtRNA that encodes leucine. Mitochondrial genome defects also include Kearns-Sayre syndrome, caused by large-scale rearrangement of mitochondrial DNA (Karaa and Goldstein, 2015) and many other site changes caused by diabetic endocrine diseases. The latter includes diabetes caused by autosomal recessive mutations in the nuclear genes Polg, RRM2B, OPA1 and MPV17 (Hopkins et al., 2010; Garone et al., 2012; Chow et al., 2017). In pancreatic B cells, mitochondria connect glucose metabolism with insulin exocytosis, thus ensuring strict control of glucose-stimulated insulin secretion (GSIS) (Casimir et al., 2009). Defects in mitochondrial function destroy this metabolic coupling and eventually promote apoptosis and pancreatic B cell death (Haythorne et al., 2019). Before the discovery of mitochondrial diabetes, other systemic diseases caused by mitochondrial defects were also known (Schaefer et al., 2013). Examples include hearing loss in immunoglobulin deposition disease, retinopathy, renal failure, and left ventricular hypertrophy without hypertension (Majamaa-Voltti et al., 2002; Whittaker et al., 2007).

At present, mitochondrial uncoupling agents have great potential in treating diseases. In mitochondria, the final step in the oxidation of substrates is the transfer of electrons from the respiratory chain to oxygen to form water. The respiratory chain uses the energy released to pump protons out of the mitochondria. Most of these protons re-enter *via* the enzyme ATP synthase and the energy is used for the synthesis of ATP. However, if protons re-enter in any other way, the mitochondria are considered to be uncoupled (Nedergaard et al., 2005).Mitochondrial uncoupling agents can reduce the degree of mitochondrial coupling and reduce insulin resistance in various tissue types, thus treating diabetes (Nedergaard et al., 2005). The first mitochondrial uncoupling agent, 2,4-dinitrophenol (DNP), was used for weight loss (Hoch and Hogan, 1973), but it was discovered that its lethal side effects were hyperlactic acidaemia and hyperthermia. Therefore, how to eliminate the side effects of mitochondrial uncouplers and retain their function to improve insulin sensitivity is an important research issue. It was found that the toxicity of 2,4-dinitrophenol decreased after modification by pyruvate dehydrogenase (PDH). The study of mitochondrial uncoupling agents provides a new idea for the treatment of type 2 diabetes (Erion et al., 2013), nonalcoholic steatohepatitis and metabolic syndrome (Ratziu et al., 2010).

### Mitochondria and Urinary System Diseases

The kidney is an organ with high energy demand and rich mitochondria. Many components of mitochondria are essential to preserve mitochondrial health and the optimal energy producing capacity of the kidney (Han et al., 2008). In the kidney, mitochondrial disorders can lead to various forms of kidney diseases, such as tubulopathy, tubulointerstitial nephritis, cystic kidney disease, or glomerular disease and most commonly focal segmental glomerulosclerosis (FSGS) (Schijvens et al., 2020). FSGS caused by defects in the coenzyme Q10 biosynthetic pathway and secondary to the mtDNA3243a > G mutation can be repaired by oral coenzyme Q10 supplementation (Hidalgo-Gutiérrez et al., 2021). In addition, a subset of miRNAs was found to be localized in human mitochondria, while many studies have indicated that miRNAs play regulatory roles in the progression of chronic kidney disease (CKD) (McClelland et al., 2015; Badal et al., 2016). For example, miR-21 is significantly upregulated in patients with a variety of kidney diseases such as renal fibrosis, septic kidney injury, and in animal models of chronic kidney disease (McClelland et al., 2015). Forced expression of miR-93 prevents the progression of diabetic nephropathy *via* mitogen and stress-activated kinase 2-mediated epigenetic regulation (Zhong et al., 2013). In addition, CoQ not only transfers electrons in the respiratory chain but is also a key antioxidant and a regulator of apoptosis as well as a cofactor for several other dehydrogenases (Doimo et al., 2014; Opalińska and Meisinger, 2015). Therefore, mitochondrial DNA mutation and CoQ10 deficiency often lead to renal insufficiency (Desbats et al., 2015).

Currently, treatment for kidney diseases associated with mitochondrial phagocytosis is mainly targeted at mitochondrial dynamics, mitochondrial phagocytosis, and mitochondrial biogenesis (Mukhopadhyay et al., 2012; Broome et al., 2021). Mitochondria-targeted antioxidants, such as MitoQ, contain an ubiquinone fraction. MitoQ provides targeted protection against exercise-induced oxidative stress by adding a lipophilic triphenylphosphine cation to the mitochondria (Broome et al., 2021). New strategies, such as prokaryotic transplantation, a mitochondrial substitution technique, may also represent potentially valuable methods in some diseases, but their related ethical issues should not be ignored (Wrigley et al., 2015).

### Mitochondria and Cancer

Mitochondria are closely associated with cancer aggressiveness and therapeutic effects. Tumour cells often evade cell death through mitophagy. Mitophagy-related liver cancer stem cells (LCSCs) are one of the key contributors to hepatocarcinogenesis, progression and recurrence (Mittermeier et al., 2020). Researchers have shown that the mitophagy receptor protein FUNDC1 promotes liver tumorigenesis upon specific knockout in the liver. FUNDC1 deletion causes damaged mitochondria to accumulate in the liver in hepatocytes, and a large amount of mtDNA is released from the mitochondrial matrix into the cytosol to activate inflammasomes. In summary, the resulting deletion of FUNDC1 leads to a decrease in mitophagy and promotes liver tumorigenesis. Overactivated inflammasomes produce a large amount of inflammatory cytokines, thus activating downstream signalling pathways such as JAK/STAT and NF-κB, which may promote the excessive proliferation of hepatocytes and eventually lead to the occurrence of liver cancer (Li et al., 2019). Mitochondria are also directly involved in the process of apoptosis. The sensitivity of cancer cells to apoptotic stimulation signals directly affects the effect of cancer treatment (Lopez and Tait, 2015). Moreover, Yasuhito Onodera et al. (Onodera et al., 2018) studied the molecules involved in the fusion and fission of mitochondria in highly invasive breast cancer cells and revealed that the fusion of mitochondria produced excessive ROS, causing cancer cells to die, while the fission of mitochondria caused cancer cells to become resistant to ROS-related cancer treatments.

Many studies have shown that mitochondrial fission and fusion are out of balance in cancer cells. There is often an increase in fission activity and a decrease in fusion activity, leading to mitochondrial fragmentation in cancer cells. Restoration of mitochondria to a confluent state by experimental means can cause damage to cancer cell growth, demonstrating that alterations in the balance of mitochondrial fission and fusion are important for tumorigenesis (Lee et al., 2014; Mishra and Chan, 2016). In addition, mitochondria are the hub of the intracellular metabolic response, which promotes the reprogramming of cancer cells through different mechanisms, resulting in changes in sugar metabolism, amino acid metabolism, fat metabolism and other aspects of cancer cells to meet the needs of the rapid growth of cancer cells (Martinou and Youle, 2011). Mutations in some genes in the mitochondrial genome are able to alter the course of tumorigenesis. Certain mutated enzymes are capable of catalysing the production of carcinogenic metabolites, enhancing the effect on chromosome structure and promoting carcinogenesis in cells. In summary, mitochondrial biology and tumorigenesis signalling networks intersect and influence each other at multiple levels.

## Conclusion

In this review, we discussed the central position of mitochondria in various cell death modes in detail. Mitochondrial oxidative stress can regulate various types of RCD, and these processes are closely linked. However, mitochondrial dysfunction can cause abnormalities in multiple organs and systems. We categorized diseases according to systems of the body and focused on the relationship between mitochondria and disease pathogenesis. Therefore, the regulation of mitochondria has great clinical potential in various diseases. Many drugs targeting the mitochondria to regulate cell death to treat disease are being researched and developed. An in-depth understanding of mitochondrial dynamics will help us gain insight into the pathogenesis of many diseases and the development of related drugs.
